# Vocabulary comprehension in adults with fragile X syndrome (FXS)

**DOI:** 10.1186/s11689-019-9285-x

**Published:** 2019-10-16

**Authors:** Anne Hoffmann, Sue Ellen Krause, Joanne Wuu, Sue Leurgans, Stephen J. Guter, Sandra S. Block, Jeff Salt, Edwin Cook, Dominick M. Maino, Elizabeth Berry-Kravis

**Affiliations:** 1 Departments of Pediatrics and Communication Disorders and Sciences, Rush University, 600 N. Paulina, 1016A AAC, Chicago, IL 60612 USA; 2 Krause Speech and Language Services, 233 E. Erie Street, Suite 815, Chicago, IL 60611 USA; 3 Department of Neurology, University of Miami Miller School of Medicine, 1120 NW 14th Street, Rm 1345, Miami, FL 33136 USA; 4 Departments of Neurological Sciences, Rush University, 1750 West Harrison, Chicago, IL 60612 USA; 5 University of Illinois at Chicago, Institute for Juvenile Research, 1747 W. Roosevelt Road, Room 155, Chicago, IL 60608 USA; 6 Illinois College of Optometry, 3241 S. Michigan Avenue, Chicago, IL 60616 USA; 7 Have Dreams, 515 Busse Highway, Suite 150, Park Ridge, IL 60068 USA; 8 Department of Psychiatry, University of Illinois at Chicago, 1747 W. Roosevelt Road, Room 155, Chicago, IL 60608 USA; 9 Departments of Pediatrics, Neurological Sciences and Biochemistry, Rush University, 1725 W. Harrison Street, Suite 710, Chicago, IL 60612 USA

**Keywords:** Fragile X syndrome, Language, Vocabulary, Comprehension, Cognition, Adults

## Abstract

**Background:**

Receptive and expressive vocabulary in adult and adolescent males with fragile X syndrome (FXS) have been shown as significantly lower than their chronological age; however, receptive vocabulary has been considered a strength relative to mental age. This has not been formally examined, however, and data are needed to compare receptive vocabulary with other language skills and with mental age in individuals with FXS. This is especially important as vocabulary measures are sometimes used as a proxy to estimate language ability.

**Methods:**

This preliminary study examined receptive vocabulary, global language, and cognitive skills in 42 adults (33 males and 9 females) with FXS as a portion of the baseline evaluation prior to randomization in a clinical trial of ampakine CX516. The battery of standardized tests addressed receptive vocabulary with the Peabody Picture Vocabulary Test, Third Edition (PPVT-III), receptive and expressive language (termed henceforth as global language) via the Preschool Language Scale, Fourth Edition or the Clinical Evaluation of Language Fundamentals, Third Edition, and non-verbal cognition via the Stanford-Binet Intelligence Scales, Fourth Edition (SB-IV).

**Results:**

Results showed (1) significantly higher receptive vocabulary than global language, (2) significantly better receptive vocabulary than non-verbal cognition, (3) equivalent non-verbal cognition and global language, and (4) severity of autism symptomatology was not correlated to receptive vocabulary or global language once non-verbal cognition was removed as factor. The scores from the PPVT-III did not represent the global language skills in our sample of adults with FXS.

**Conclusions:**

Findings from this investigation strongly suggest that the PPVT-III should not be used as a screening tool for language levels or cognitive function in clinical studies since the scores from the PPVT-III were not representative of global language or non-verbal cognitive skills in adults with intellectual disabilities. This finding is critical in order to understand how to evaluate, as well as to treat, language in individuals with FXS. Development of efficient and appropriate tools to measure language, cognition, and behavior in individuals with FXS is essential.

## Background

FXS is the most common cause of inherited intellectual disability with a prevalence of 1/4000 males and 1/5000–8000 females [1]. The behavioral phenotype of FXS includes cognitive deficits, hyperactivity, attention disorders, anxiety and mood instability, tactile defensiveness and hypersensitivity to sensory stimuli, and patterns of behavior consistent with autism spectrum disorder (ASD) [2]. Females with FXS often have an overall milder presentation of the phenotype due to the second “protective” X chromosome. About 50–60% of males and 20% of females with FXS meet criteria for an ASD [3]. Language comprehension and expression deficits, including pragmatics, as well as speech disorders affecting phonology, speech prosody, and speech fluency occur in varying degrees [5–7].

Research on syndrome specific language profiles often benefits from clear delineation of types of language. For the purposes of this study, we will be examining receptive vocabulary as it compares to receptive and expressive language more broadly. Receptive vocabulary refers to those words that an individual is able to comprehend and is limited in scope to the language domain of content. Receptive and expressive language is a broader term, and for this study will be defined as encompassing the two language domains of content as well as form (referring to morphosyntactic comprehension and production). We will be referring to this combination of receptive and expressive language as global language for succinctness. Both research and clinical practice have used vocabulary measures to estimate overall language [4, 7]. However, as we will show below, this practice has been shown as inaccurate in children and adolescents with FXS and we will extend that research to adults with FXS.

### Language in FXS

Early research on males with FXS showed that they perform well below their chronological age (CA) on measures of receptive and expressive vocabulary [9–11]. Adult males with FXS have demonstrated vocabulary scores similar to those of age- and cognitively-matched males with nonspecific forms of intellectual disability and with autism [10, 12]. Receptive vocabulary in a familial sample of adult males with FXS on the Peabody Picture Vocabulary Test-Revised (PPVT-R [13]) exceeded non-verbal mental age (MA) [9]; however, when Abbeduto et al. [15] studied receptive language in adolescents and young adults with FXS via the Test for Auditory Comprehension of Language-Revised (TACL-R [14]), receptive vocabulary was commensurate with non-verbal MA based on the Word Classes and Relations (WC&R) subtest scores.

Less is known about vocabulary development in females with FXS. Abbeduto et al. [15] found that females performed significantly better than males on the TACL-R. Moreover, the difference between the age-equivalent scores on the TACL-R and non-verbal MA was not significant across sex, yielding similar profiles. More recently, 7–15 year-old girls with full mutation FXS have shown receptive vocabulary as measured by the Peabody Picture Vocabulary Test, Third Edition (PPVT-III [20]) or the Peabody Picture Vocabulary Test, Fourth Edition (PPVT-4 [21]) as an area of strength, albeit with considerable variability, relative to non-verbal cognition [22].

Vocabulary abilities in varied populations of individuals with intellectual disability have been shown as divergent from other language functions and cognitive skills. For instance, significantly better receptive vocabulary than syntax has been documented in individuals with Down syndrome (DS) [15, 16], but not in individuals with FXS across subtests of the TACL-R [15]. Children with Williams syndrome have shown comparatively better receptive vocabulary than non-verbal reasoning [17]. Roberts et al. [19] reported that boys with DS demonstrated lower receptive vocabulary on the PPVT-III and expressive vocabulary on the Expressive Vocabulary Test (EVT [28]) than non-verbal cognitively matched boys with typical development. Consistent with Chapman, Seung, Schwartz, and Kay-Raining Bird’s [18] study of children and adolescents with DS, Roberts et al. [19] found that expressive vocabulary in boys with DS was lower than for typically developing children matched for MA. Finestack, Sterling, and Abbeduto [29] showed no significant group differences between 10 and 16-year-old individuals with FXS and non-verbal cognitively age-matched typically developing individuals in receptive vocabulary as measured by the PPVT-III and a measure of receptive grammar as measured by the Test for Reception of Grammar-Version 2 (TROG-2 [27]). Moreover, receptive and expressive vocabulary, the latter of which was measured by the Expressive Vocabulary Test-Second Edition (EVT-2 [28]), were not significantly different between these groups after controlling for non-verbal IQ.

Language ability of young males with comorbid FXS and ASD (FXS + ASD) has been shown as more impaired than in FXS males without autism ([8, 30, 32, 33]). Roberts et al. [19] reported that after adjusting for non-verbal cognitive age, boys with FXS did not differ in receptive and expressive vocabulary from typically developing boys. However, boys with FXS + ASD performed lower than the cognitively matched group in expressive vocabulary. Philofsky et al. [30] found that children with FXS + ASD performed worse than children with single diagnoses of either FXS or ASD on receptive and expressive scales of the Mullen Scales of Early Learning (Mullen [31]), which include measures of vocabulary. Haebig and Sterling [34] studied boys with ASD and ASD + FXS and found that non-verbal IQ was a significant predictor of both receptive vocabulary (PPVT-4) and expressive vocabulary (EVT-2) in both groups, but contrary to their prediction, autism severity scores did not predict vocabulary scores.

Morphology refers to the rules governing how words are formed, while syntax refers to the rules determining how sentences are formed. In these areas, individuals with FXS typically show stronger receptive than expressive language with receptive language skills below CA but commensurate with non-verbal MA [12, 35]. Individuals with FXS + ASD show receptive language skills falling below their non-verbal MA with increasing severity as the ASD increases [8, 32, 33, 35]).

### Assessment of language in FXS

Evaluation of language abilities in individuals with FXS is challenging given the population heterogeneity. Verbal and non-verbal abilities cover a broad range, and existing, norm-referenced tests do not offer the necessary level and type of language tasks to appropriately target all individuals at a given CA. Moreover, participant sample sizes of many studies available have been limited due to the rarity of the condition (e.g., [15, 23]).

Thus, different findings concerning vocabulary and language in general are partly attributable to differences in measures used across studies and the particular cohort studied. The PPVT is considered a classic measure of receptive vocabulary ability and assesses many more items than the WC&R Subtest (TACL-R) or the Vocabulary subtest, Test for Auditory Comprehension of Language, Third Edition (TACL-3 [24]). Moreover, the point has been made that the PPVT assesses a lexical bank whereas the TACL taps into language concepts, which offer greater challenge [17, 26]. In fact, the PPVT has yielded higher scores than the TACL-3 Vocabulary subtest for adolescents with DS and with intellectual disability of unknown origin ([25, 26]). Data are needed to compare receptive vocabulary with other language skills and with non-verbal MA in adults with FXS to contribute to the descriptive profile of adults with intellectual disability. An understanding of the language profile in adults with FXS is crucial for the delivery of language interventions. Moreover, developing a valid and reliable battery of language and cognitive assessments is critical for clinical trials research with FXS and other groups of individuals with intellectual disability. This is especially true as some studies have used receptive vocabulary assessments as a proxy for verbal cognition [37]. As previous research has demonstrated the appearance of a decline in IQ for individuals with FXS as they transition to adolescence and adulthood, one cannot assume that patterns present in younger groups remain stable in adulthood [36].

The purpose of this preliminary study was to compare receptive vocabulary to a measure of global language and to non-verbal cognition in a relatively large sample of adults with FXS who spanned a broad spectrum of cognitive and language competencies. Specifically, we investigated the following research questions:

1. Is receptive vocabulary significantly different than global language?
2. Is receptive vocabulary significantly different than non-verbal cognition?
3. Is global language significantly different than non-verbal cognition?
4. What is the role of ASD in these comparisons?

Our hypotheses were that receptive vocabulary would be a relative strength as compared to global language and non-verbal cognition, that global language and non-verbal cognition would be similar, and that the ASD diagnosis would not affect this pattern.

## Methods

### Participants

The participant cohort consisted of 43 adults with FXS; however, one individual was eliminated from the analyses as he was unable to complete standardized testing. This left 42 adults with FXS and language/cognitive data that could be analyzed for this study (33 males and 9 females), ages 18–49 years with a mean age of 26.4 years (SD = 8.2 years). Of these individuals, 39 were Caucasian, 2 were African American, and 1 was Hispanic. Informed written consent was obtained from either the participant or the parent/legal guardian prior to participation, and assent was obtained from each participant who was not his/her own legal guardian. The study was approved by the Institutional Review Board at Rush University Medical Center.

Language and cognitive testing utilized for the analyses in the present study was obtained from all participants as a portion of the baseline evaluation prior to randomization in a clinical trial of CX516 conducted between 2002 and 2004 [38]. Eligibility criteria for the trial included the following: (1) 18 to 50 years of age; (2) an expansion mutation in FMR1 with at least partial methylation (full mutation), consistent with a diagnosis of fragile X syndrome by DNA analysis; (3) intellectual disability with measured IQ between 20 and 85; (4) normal hearing sensitivity and normal visual acuity (with best correction); (5) stable medication regimen for 8 weeks prior to enrollment; (6) negative pregnancy test (female participants) at enrollment; and (7) native English speaker. Participants were excluded if they had (1) a recent history of seizure, epilepsy, or syncope or any possible clinical seizure in the past 5 years; (2) an unresolved medical issue impacting performance; (3) behavioral dysfunction sufficiently severe to preclude cooperation with testing; or (4) other medically related issues that might potentially impact the clinical trial.

### Materials and procedures

All participants were tested individually on a battery of standardized measures. Measures used were the versions of tests available at the time the study was done. Included were the Stanford-Binet, Fourth Edition (SB-IV [40]) PPVT-III, Form A, the Autism Diagnostic Observation Schedule (ADOS [41]), and a measure of global language. For the purposes of this study, the Abstract Visual Reasoning composite from the SB-IV was used to determine mental age (MA), as it is considered to best correspond to non-verbal IQ [39]. The ADOS is a standardized, semi-structured, assessment of interaction, communication, repetitive, and stereotyped behaviors. This instrument is considered a gold-standard diagnostic instrument for autism diagnosis and was used to determine autism status [42].

The PPVT-III is a four-alternative forced-choice paradigm, depicting the target stimuli and foils in black and white line drawings. Either the Preschool Language Scale, Fourth Edition (PLS-4 [43]), for those participants with a MA of less than 6 years, 0 months (< 6;0, *n* = 26), or the Clinical Evaluation of Language Fundamentals, Third Edition (CELF-3 [44]), for participants with a MA of greater than 6 years was administered. Fourteen participants with a MA of 6;0–8;11 and two participants with a MA of ≥ 9;0 were tested on the respective forms of the CELF-3. The PLS-4 and respective forms of the CELF-3 were selected such that the MA of our participants corresponded to the CA of individuals in the tests’ standardization procedures. The PLS is comprised of two subtests—Auditory Comprehension (AC) and Expressive Communication (EC). The AC subtest includes colorful pictured stimuli and common objects either of which is presented with a spoken stimulus addressing a range of skills from simple auditory-verbal attention tasks to basic vocabulary, language concepts, syntax, concrete, and inferential reasoning. The EC subtest is comprised of spoken stimuli both with and without colorful pictured stimuli and tapping into expressive vocabulary, syntax, and morphology in sentence completion and spontaneous speech, completion of verbal analogies, narrative recitation, and a phonological awareness task. The CELF (6;0–8;11) addresses receptive language in three separate subtests for syntax, language concepts embedded in spoken directions, and word classes. The CELF (6;0–8;11) addresses expressive language via three separate subtests for syntax and morphology in sentence completion, sentence recall, and sentence formulation. The CELF (≥ 9;0) assesses comprehension in three subtests for language concepts embedded in spoken directions, word classes, and semantic relationships. The three expressive language subtests (CELF ≥ 9;0) include sentence formulation, sentence recall, and sentence assembly. The overall language measures for our subject sample spanned a range of skills pertinent to the range of cognitive levels as demonstrated in our subject sample. The same examiner (second author) tested each participant for the PPVT-III and PLS-4 or CELF-3. The PPVT-III was consistently administered before the PLS-4 or CELF-3. All but two participants were tested by the same examiner for the SB-IV; those two were tested by the same alternative examiner. Each participant was seated comfortably in a quiet room with the examiner in the Fragile X Clinic area at the Rush University Medical Center.

### Statistical analysis

To maintain consistency between the PLS-4 and CELF-3, and comparability across receptive vocabulary and non-verbal cognitive tests, the AE scores for each test were utilized. Such developmental scores are derived by identifying the age group that has a mean score closest to that received by the study participant. The AE score does not reflect normal group variability [45]. However, the application of standard scores based on the MA of our participants would yield inadequate discrimination of standard scores at the lower end of the ability level distribution (i.e., flooring effect). For example, 24 of the participants scored at floor on the SB-IV. This is an unfortunately common challenge when testing individuals with intellectual disability and one which is the focus of ongoing efforts to improve current outcome measures [46–49]. Considering the preliminary nature of this and the parent study, the AE scores provided a consistent manner in which to compare scores on all measures administered, although the use of AE scores is a clear limitation.

Initial analysis of the data using a Shapiro-Wilk test revealed that the global language AE scores for the males violated the assumption of normality (*p* < .05). Thus, non-parametric measures were used for analyses.

On the CELF-3, an AE score is only available on the Total Test. The raw scores from the comprehension component of the CELF-3 strongly correlated with the Total AE (*r =* 0.91, *p* < 0.0001), implying that the Total AE from the CELF-3 reflects performance on the comprehension component. For the PLS-4, the AEs of the Total Score, the Auditory Comprehension subtest, and the Expressive Communication subtests were also highly correlated (*r =* 0.80, *p* < 0.0001), and all three showed very similar relationships with the PPVT-III. We therefore chose the Total PLS-4 AE in our analysis, so that we could combine it with the Total AE scores from the CELF-3 to yield a range of functional language levels from “youngest” to “oldest.” AE scores were compared between tests via Wilcoxon signed-rank tests and their agreement assessed by Spearman’s rho rank correlations. The level of statistical significance was set at 0.05 (two-sided). For analyses, participants were divided by sex to ensure that the trend for stronger performance on language and IQ assessments by females with FXS did not drive the results. To further assess the role of cognition, analyses were also performed with the participants grouped by mental age, with comparisons between groups made using Mann-Whitney *U* tests.

The role of ASD symptomatology was addressed by using calculated severity scores (CSS) from the ADOS. Of the participants, 35 had the full ADOS protocol available for analysis. Each of these participants had been assessed using Module 4, indicating that they were adults with fluent speech (fluent speech defined as a range of flexible sentence types that make some reference outside the immediate context [41]). Raw scores were used to calculate severity scores using the algorithm developed in Hus and Lord [50], with higher scores being indicative of increased severity. Correlations between the CSS and performance on the global language measure and the PPVT-III were run with non-verbal MA partialed out to avoid potential confounding with increased intellectual disability.

## Results

A summary of the means, standard deviations, and ranges of AE scores for the PPVT III, PLS-4/CELF-3, SB-IV, SB-IV Abstract/Visual Reasoning, and CSS for the ADOS is provided in Table 1. Data from 42 participants (9 females, 33 males) were analyzed for comparisons between the PPVT-III and SB-IV Abstract-Visual Reasoning. Data from 38 participants (8 females, 30 males) were analyzed for the comparisons between SB-IV Abstract/Visual Reasoning and PLS-4/CELF-3 because of incomplete data for 6 participants. However, comparison of group means between SB-IV Abstract/Visual Reasoning standard scores for the missing participants and the overall group means showed similar performance (for males, *m*_group =_ 45.43 vs. *m*_missing_ = 46.00, and for females, *m*_group =_ 68.5 vs. *m*_missing_ = 72), so it is likely the same patterns would have been observed if that AE scores for those participants had been available.

**Table 1:** Mean and range of age-equivalent (AE) and calculated severity scores (CSS) on indicated tests

		Number	Mean AE + SD	Range
PPVT-III	Males	33	7.12 + 3.32	2.00–18.17
	Females	9	12.79 + 3.03	8.50–17.00
	All	42	8.4 + 4.0	2.0–18.2
PLS-4/CELF-3	Males	33	4.55 + 3.08	3.08–5.08
	Females	9	6.62 + 2.06	4.75–10.92
	All	42	5.3 + 1.8	3.1–10.9
SB-IV Abstract/Visual Reasoning	Males	28	4.55 + 1.57	2.42–8.75
	Females	8	7.95 + 1.50	5.33–9.67
	All	36	5.3 + 2.10	2.42–9.67
		Number	Mean CSS + SD	Range
ADOS	Males	25	17.08 + 5.15	7–25
	Females	9	8.83 + 1.58	5–10
	All	35	14.83 + 5.92	5–25

*PPVT-III* Peabody Picture Vocabulary Test, Third Edition, Form A, *PLS-4* Preschool Language Scale, Fourth Edition, *CELF-3* Clinical Evaluation of Language Fundamentals, Third Edition, *SB-IV* Abstract/Verbal Reasoning Composite of the Stanford-Binet Intelligence Scales, Fourth Edition, *ADOS* Autism Diagnostic Observation Schedule

### Relationships between receptive vocabulary, global language, and non-verbal cognition

Investigation of the comparative performance between vocabulary comprehension (PPVT-III) and global language (PLS-4/CELF-3) using Wilcoxon signed-rank tests revealed that AE scores on the PPVT-III were significantly higher than those on the PLS-4/CELF-3 for both females (*r =* − .62*, p <* 0.001) and males (*r =* − .52*, p <* 0.001) indicating better receptive vocabulary than global language. Correlations were run to determine if there was a factor associated with a larger difference between receptive vocabulary and global language. For males, there was a correlation between non-verbal MA and a larger receptive vocabulary/global language discrepancy (*r =* .45, *p* = .015,), thus indicating that as non-verbal MA increased, receptive vocabulary outpaced global language skills more significantly. For females, there was a correlation between larger receptive vocabulary/global language discrepancy and age (*r =* .927*, p <* .001). Comparison of receptive vocabulary scores with the non-verbal cognitive scores revealed that AE scores on the PPVT-III were also significantly higher than AE scores on the SB-IV Abstract/Visual Reasoning for both females (*r =* − .63*, p <* .001) and males (*r =* .51*, p <* .001), suggesting stronger receptive vocabulary than non-verbal cognition.

**Fig. 1:**
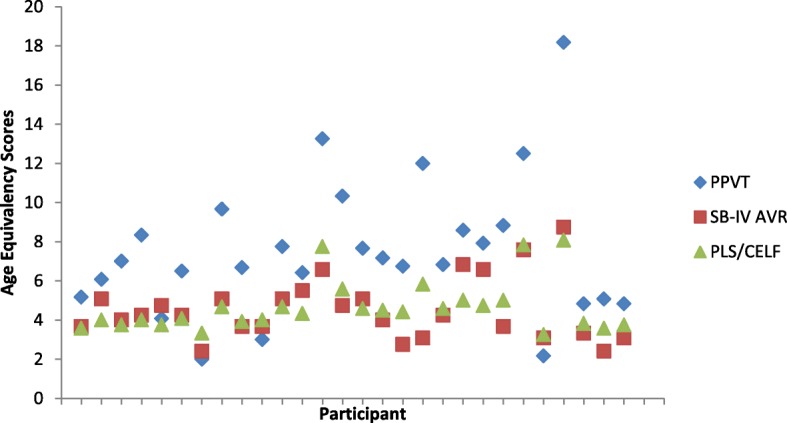
Age-equivalent scores in years for male participants

**Fig. 2:**
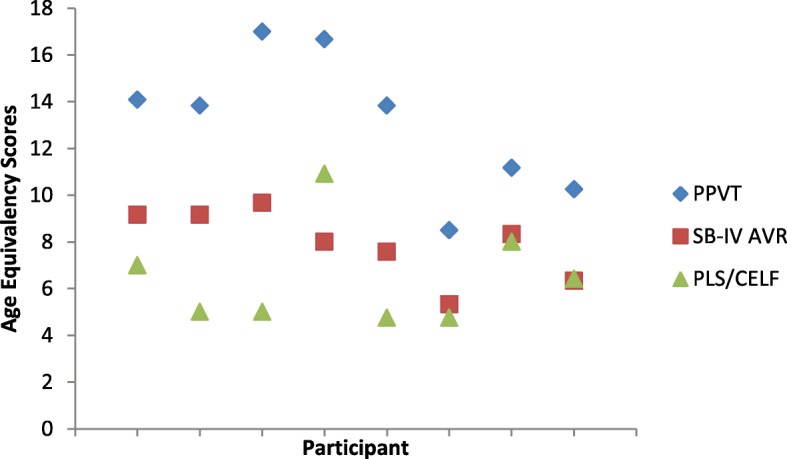
Age-equivalent scores in years for female participants

Further comparison of global language with non-verbal cognition demonstrated that the AE score on the PLS-4/CELF-3 did not differ significantly from the AE on the SB-IV Abstract/Visual Reasoning domain for either females (*r =* − .385*, p =* .196) or males (*r =* − .075*, p =* .346), suggesting that the non-verbal cognition is at a similar level of ability as language use. The relationship between the three different AE scores is further illustrated in Figs. 1 and 2.

### Relationships between global language and receptive vocabulary grouped by non-verbal mental age

To ensure that the results did not differ across the wide range of mental ages represented in the participants, additional analyses were also performed with participants divided into groups based on mental age: group 1 included 11 participants whose non-verbal MA ranged from 2.42–3.67 (*m* = 3.17), group 2 included 13 participants whose non-verbal MA ranged from 4.00–5.50 (*m* = 4.72), group 3 included 7 participants whose non-verbal MA ranged from 6.33–7.58 (*m* = 6.87), and group 4 included 6 participants whose non-verbal MA ranged from 8.0–9.67 (*m* = 8.85). Data were not available for 6 participants. For the non-verbal MA analyses, males and females were not separated as the groups were already small and the role of cognition was the focus.

Mann-Whitney *U* tests were used to compare average difference between PPVT-III AEs and global language AEs (i.e., the global language AE subtracted from the PPVT-III AE). Group 1 and group 2 both had means that were significantly lower than the mean for group 3 (group 1 *p* < .05, *U =* 8.5; group 2 *p* < .05, *U =* 14.0) and for group 4 (group 1 *p* < .05, *U =* 3; group 2 *p* < .05, *U =* 5). Group 1 and group 2 were not significantly different from each other (*p* = .183, *U =* 48.5) nor were group 3 and group 4 (*p* = .1, *U =* 9.5). In other words, the PPVT-III AE scores tended to be more discrepant from the global language AE scores for those individuals with a higher MA, with the PPVT-III being higher than the global language.

### Relationships between autism symptomatology and language/cognitive ability

Analyses were also performed to assess the role that autism symptomatology might play in language and cognitive ability. Using the Calculated Severity Score (CSS) from the ADOS, for the males, higher severity scores were associated with lower PPVT-III AE scores (*r =* − .478 *p* < .05), lower global language scores (*r =* − .710, *p* < .001), and lower non-verbal MA (*r =* − .705, *p* < .001). To remove the possible confounding factor of IQ, correlations were run a second time with non-verbal MA partialed out. With this alteration, there was no longer a correlation between the CSS and either PPVT-III AE scores (*r =* .126*, p =* .586) or global language scores (*r =* .022, *p =* .926). For females, there were no significant correlations between autism symptomatology and performance on any measures, nor between autism symptomatology and the discrepancy between receptive vocabulary and global language. This was also true when non-verbal MA was partialed out. However, the group of females with FXS was very small and generalization of these results is limited.

## Discussion

Receptive vocabulary in our sample of adults with FXS was superior to global language, verbal reasoning, and non-verbal cognition. Receptive vocabulary as measured by the PPVT-III was not a representative benchmark of expressive or receptive language skills in our pilot study. However, these global language skills were consistent with non-verbal MA as measured by the SB-IV, suggesting that receptive vocabulary may be a relative strength with this older population of individuals with FXS. This was true for both the female and male participants, which is the first time this has been studied in females with FXS. This suggests that the relative strength of receptive vocabulary may be a characteristic of FXS in general and not associated with a specific sex or level of functioning. Females with FXS tend to have higher levels of functioning then males in general; this was true for our participant sample, as well. This disparity was reflected in the CELF-3 data, as 9/9 females had sufficient language to be assessed with this measure, while only 6/33 males did. The discrepancy between receptive vocabulary and global language was increased with higher non-verbal MA for males, possibly secondary to increased ability to access and comprehend more vocabulary items. The correlation between age and increased discrepancy between receptive vocabulary and global language for females is likely reflective of the increased exposure to vocabulary that comes with time.

The role of ASD in FXS is still problematic. Analyses revealed that increased autism symptomatology on the communication subscale was correlated with lower scores in receptive vocabulary, global language, and non-verbal cognitive skills. This supports previous findings that individuals with more autistic features tend to score on the lower end of measures of language and cognition [22]. However, when non-verbal MA was partialed out, the correlation vanished. With non-verbal MA partialed out, there was also no correlation between ASD severity and the size of the gap between receptive vocabulary and global language. This suggests that while increased ASD severity is associated with more cognitive impairment, it is not as strongly linked to the pattern of language impairment associated with FXS.

Our preliminary study of adults with FXS posed a particular challenge with test selection, since the range in participants’ abilities varied widely across our sample. The PLS-4, which is weighted toward measuring semantic like skills, was appropriate for the lower functioning participants. The CELF-3 is more balanced across areas of language by the inclusion of more varied syntactic components. It was not possible to offer the same measure to such a divergent group of participants. These test versions, as well as the SB-IV, were the most updated at the time of data collection. Although they have since been revised, it is unlikely that our data would have been impacted by the changes. Moreover, while there are limitations in using AE scores, the use of AE scores was the only practical way to evaluate relative performance in our pilot data. While more recent versions of these assessments provide more psychometrically sound means of assessing progress within a measure (e.g., growth scale values), there is still no method of comparing scores between measures for the majority of assessments without resorting to AE scores. This practice is often necessary when using tests normed on the general population while studying populations with intellectual disability, who are more likely to have floor effects that invalidate standard scores.

The finding of the participants in this study to have significantly higher receptive vocabulary scores than both receptive/expressive language and non-verbal cognition highlights the danger of using assessments such as the PPVT as proxies for language assessments. This has been documented in other groups with language impairment [51], and the extension of this finding to individuals with FXS suggests that this practice may be inappropriate for other groups as well.

### Limitations

The study of individuals with FXS highlights the demand for developing a fair representation of the spectrum of language skills considering the documented comorbid traits. Future investigation may delineate factors that contribute to the variance by using comparative groups of cognitively age-matched typically developing and cognitively impaired individuals with varied phenotypes. The group matched designs could also clarify the measurement dilemma encountered with cognitively impaired study participants [52]. Such differentiation exceeded the limits of this investigation, which involved a sample of convenience for a pilot study. The ability to engage in assessment for extended periods of time complicates the selection of appropriate and valid measures for individuals with intellectual impairment, which is underscored in individuals with FXS, especially when coupled with the lack of appropriate measures normed for individuals in the age range represented by our sample. Therefore, development of efficient and appropriate tools to measure cognition, language, and behavior in individuals with intellectual disability, including those with FXS is essential.

## Conclusions

Findings from this investigation strongly suggest that the PPVT should not be used as a screening tool for language levels or cognitive function in clinical studies since the scores from the PPVT-III are not representative of global language or cognitive skills in adults with FXS. The finding of better receptive vocabulary than global language and non-verbal cognition is critical in order to understand how to evaluate and treat language deficits in individuals with FXS.

## Data Availability

The data sets from the current study are available from the corresponding author upon reasonable request.

## Abbreviations

ADOS: Autism Diagnostic Observation Schedule
AE: Age-equivalent
CA: Chronological age
CSS: Calculated severity scores
FXS + ASD: FXS and ASD
FXS: Fragile X syndrome
IQ: Intelligence quotient
MA: Mental age
PLS-4: Preschool Language Scale, Fourth Edition
PPVT-4: Peabody Picture Vocabulary Test, Fourth Edition
PPVT-III: Peabody Picture Vocabulary Test, Third Edition
PPVT-R: Peabody Picture Vocabulary Test-Revised
SB-IV: Stanford-Binet Intelligence Scales, Fourth Edition
SB-IV-Verbal: Verbal Reasoning subtest of the SB-IV
SD: Standard deviation
TACL-3: Test of Auditory Comprehension of Language, Third Edition
TACL-R: Test of Auditory Comprehension of Language-Revised
TROG-2: Test for Reception of Grammar, Version 2
WC&R: Word Classes and Relations
